# Engineering the electronic properties of MoTe_2_ via defect control

**DOI:** 10.1080/14686996.2024.2388502

**Published:** 2024-08-05

**Authors:** Celal Yelgel, Övgü C. Yelgel

**Affiliations:** a Department of Electricity and Energy, Recep Tayyip Erdoğan University, Rize, Türkiye; b The Computational Science and Machine Learning Laboratory, Recep Tayyip Erdoğan University, Rize, Türkiye; c Department of Electrical-Electronics Engineering, Recep Tayyip Erdoğan University, Rize, Türkiye

**Keywords:** Transition metal dichalcogenides, DFT, MoTe_2_, defect control, electronic properties

## Abstract

The remarkable electronic properties of monolayer MoTe_2_ make it a very adaptable material for use in optoelectronic and nano-electronic applications. MoTe_2_ growth often exhibits intrinsic defects, which significantly influence the material’s characteristics. In this work, we conducted a thorough investigation of the electronic characteristics of intrinsic defects, including point defects, in monolayer MoTe_2_ using first-principles calculations based on density functional theory (DFT). Our findings indicate that the presence of point defects leads to the formation of n-type properties as the Fermi level situates above the conduction band. Our first-principles density functional theory calculation revealed an appearance of donor level in the band gap close to the conduction band in MoTe_2_. Our study signifies that the formation energy of a vacancy in a Te atom is lower than that of both a vacancy in a Mo atom and two vacancies in Te atom. This suggests that during the synthesis process, it is more probable for Te atom vacancies to be created. A defect in the pristine monolayer of MoTe_2_ leads to a slight decrease in the band gap, causing a transition from a direct band gap semiconductor to an indirect band gap semiconductor. The results of our study indicate that the presence of vacancy defects may modify the electronic properties of monolayer MoTe_2_, suggesting its potential as a new platform for electronic applications. Hence, our analysis offers significant theoretical backing for defect engineering in MoTe_2_ monolayers and other 2D materials, a critical aspect in the advancement of nanoscale devices with the desired functionality.

## IMPACT STATEMENT

The study explores the electronic properties of monolayer MoTe2, revealing intrinsic defects that can enhance its potential for electronic applications and providing theoretical support for defect engineering in 2D materials.

## GRAPHICAL ABSTRACT

**Figure:**
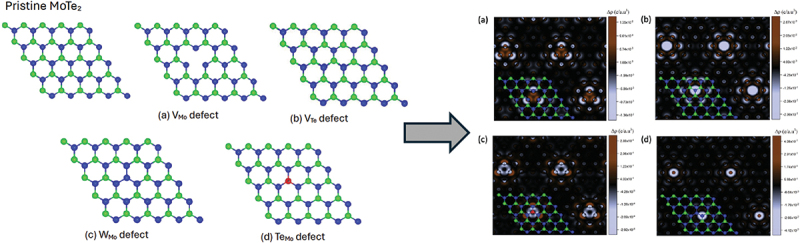

## Introduction

Since the successful synthesis of graphene, a single layered honeycomb structure of carbon atoms, by Novoselov et al. in 2004 [1], there has been steady progress in scientific interest in many further two-dimensional (2D) monolayers. Graphene has long been considered the most promising candidate to replace silicon. Nevertheless, the absence of a band gap in graphene limits its potential use in semiconductor devices. Therefore, in addition to graphene, additional single layered systems that have been widely researched include silicene, germanene, stanene, phosphorene, and other single layered transition metal dichalcogenides (MX_2_, *M* = transition metal and X = chalcogen) [2–15]. These monolayer systems, characterised by 2D structures, often display distinct features compared to their three-dimensional counterparts. As a result, these systems have become significant topics of investigation in both experimental and theoretical investigations. From an application perspective, 2D semiconducting devices possess a minimal thickness and broad band gaps, making them significant in the fields of optoelectronics, photovoltaics, spintronics, sensors, valleytronics, biomedicine, and efficient catalysis. Layered transition metal dichalcogenides (TMDCs) have attracted significant research attention among the widely studied 2D semiconductors [16–18]. This is primarily owing to their optoelectronic characteristics, including the ability to tune bandgaps and achieve valley polarisation. The investigation of doping and point defects in thin layered TMDCs received renewed attention in recent years. Currently, there is a growing interest in the telluride-based TMDCs compared to other TMDCs materials. This is largely due to their ability to exist in a wide variety of stable structural phases (2 H, 1T, and 1T′) at 300 K [19]. MoTe_2_ in its bulk form is a kind of semiconductor that has an indirect band gap [20]. However, when the thickness is reduced to a single layer, it undergoes a change and becomes a direct band gap semiconductor with a band gap of 1.10 eV due to the quantum confinement effect [21]. Therefore, monolayer MoTe_2_ is highly regarded among TMDCs due to its favourable band gap and tunability of its mobility via doping as the maximum mobility of 178.73 cm^2^/V.s obtained [22,23]. These characteristics highlight the significant potential of this fascinating material. Controlling the atomic structure has great potential for modifying electronic characteristics to create devices and enhance catalytic processes. The physical characteristics of monolayer MoTe_2_ may be further adjusted by deliberately introducing defects. Hence, a comprehensive comprehension of the influence of vacancy defects on electronic properties is crucial in determining appropriate materials that may be manipulated under certain synthesis and processing circumstances. MoTe_2_ nanosheets have been synthesised with success in experimentally [24]. It has been observed that these layered compounds can be efficiently dispersed in common solvents and can be formed into films or deposited as individual flakes. This suggests that these materials have the potential to be exfoliated into monolayers. Various polymorphs, such as the trigonal prismatic 2 H phase, the monoclinic 1T’-phase, and the orthorhombic Td-phase, may exist based on the arrangement of Te atoms [25–27]. Research has shown that the 2 H-phase of MoTe_2_ is more energetically desirable compared to the other two phases [28]. Therefore, our subsequent discussion will concentrate only on the MoTe_2_-2 H-phase. The process of exfoliating large-area monolayer TMDs has been improved by chemical vapour transport (CVT) and chemical vapour deposition (CVD) [29]. At equilibrium, the potential energy of the system consistently rises in relation to various atomic movements. Using vibrational spectra as a filter can help validate the stability of materials, as the presence of imaginary frequency indicates material instability. Theoretical computations by using first-principles calculations and experimental analysis by using broadband optical pump-probe microscopy have been recently reported on the dynamic stability and phonon transport properties of monolayer MoTe_2_ [30,31].

This advancement has expanded the range of applications for telluride-based TMDs, including logic transistors, superconductors, and valley optoelectronics [31]. Structural defects are commonly observed in large-scale single-layer materials synthesised under changing growth conditions utilising the CVD or CVT technique. Prior empirical investigations have shown the presence of several defects in the monolayer TMDs, including point defects and boundaries [32,33]. The imperfections have the potential to greatly impact the material’s geometric, mechanical, optical, thermal, and electronic characteristics. Moreover, the intrinsic structural imperfections in metal 2D TMDs provide a promising prospect for manipulating the specific characteristics in novel applications. Over the last several years, there has been a great deal studies, both experimental and theoretical, on the electronic properties of defects in materials such as MoS_2_, MoSe_2_, WS_2_, and others [20,24,29,33–38]. Nevertheless, further investigation of the electronic properties related to intrinsic defects in monolayer MoTe_2_ is still required.

Understanding the effect of defects is crucial to ensure the dependable functioning of devices. This paper presents a comprehensive analysis of the electronic structure of intrinsic defects, such as point defects and substitutional defects by replacing the Mo atom in monolayer MoTe_2_. We systematically studied the electronic and structural properties of monolayer MoTe_2_ with defects by means of first-principles calculations based on density functional theory (DFT). We calculated the defect formation energy of monolayer MoTe_2_. It is found that the lowest formation energies are 67.296 meV/atom and 15.243 meV/atom for Te vacancy and W substitutional defects. When we create additional Te defects by removing one of the upper Te atoms and one of the lower Te atoms, the formation energy increased to 125.651 meV/atom. Additionally, the substitutions of Mo site with Te and W atoms have the low formation energies comparing with Mo vacancy defect. We thoroughly explained the similarities and differences in the electronic properties of various intrinsic defects. Our results suggest that by replacing the Mo site the direct band gap in pristine monolayer MoTe_2_ can be retained at the **K** point while the band gap value is reduced to 0.112 eV by Te substitutional effect. Furthermore, it is shown that all defects studied in this work suggest a promising candidate for n-type material. These defects in monolayer MoTe_2_ have the potential to enhance the material’s characteristics and provide remarkable functions.

## Computational method

In this work, we used 5 × 5 monolayer MoTe_2_ supercell for Mo and Te vacancy defects and single W and Te substitutions. To avoid interaction between periodic images, the vacuum region of 20 Å is inserted in the out-of-plane direction. All the calculations were performed using the plane-wave based Quantum Espresso PWSCF package [39]. The generalized gradient approximation (GGA) of Perdew–Burke–Ernzerhof (PBE) was used for the exchange-correlation potential [40]. The PBE optimised in-plane lattice constant of 3.557Å for monolayer MoTe_2_ was used to generate the supercells. The spin-orbit coupling was included using fully relativistic projector-augmented-wave potentials for Mo, Te, and W atoms [41]. The total energy and Hellman-Feynman force thresholds were set to be less than 106 eV and 0.001 Ry/a.u, respectively, in our calculations. The energy cutoffs were set at 60 Rydberg (Ry) for the plane-wave basis and 480 Ry for the charge density. The Brillouin zone was sampled using the Monkhorst-Pack uniform k-grid of 6 × 6 × 1 [42]. A Methfessel-Paxton smearing of 0.005 Ry was adopted in all calculations [43]. The charge density differences were calculated using the following expression,

Δρ=ρ(Defected supercell)−ρ(Pristine supercell),

where ρ(Defected supercell) and ρ(Pristine supercell) are total charge densities for 5 × 5 supercell with Mo and Te vacancy defects and single W and Te substitutions and pristine 5 × 5 supercell, respectively.

## Results and discussion

**Figure 1.**
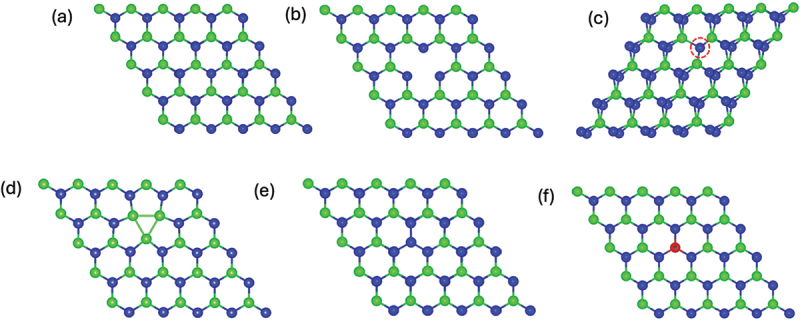
The generation of defect states in monolayer MoTe_2_. (a) The atomic structure of pristine MoTe_2_ monolayer, typical vacancy point defects; (b) V_Mo_ and (c) V_Te_ , (d) V_2Te_ and (e) the substitution of Mo atom with Te and (f) W atoms, respectively. The green and blue balls represent Mo and Te atoms, respectively. The red dotted circle represents the location of V_Te_ defect.

The monolayer 2 H – MoTe_2_ has a hexagonal crystal structure with a space group (*p*-3m1), as seen in Figure 1(a). The Mo layer is situated between the two Te layers, creating a sandwich configuration known as Te – Mo – Te. The Mo atom is surrounded by six Te atoms arranged in a trigonal prismatic coordination [44]. Before exploring into intrinsic point defects, it is essential to begin with the pristine MoTe_2_ monolayer. MoTe_2_ displays multiple-phase configurations, including 2 H, 1T, and 1T′, which are determined by the arrangement of its layers. The 2 H phase has the highest stability at normal room temperature [45]. Therefore, the subsequent discussion will be limited to the subject of 2 H-MoTe_2_. The computed lattice constant and bond length between the Mo atom and its nearest neighbour Te atoms are, a = 3.557 Å and d = 2.738 Å, respectively. These values are consistent with the previous experimental [21] and theoretical [46,47] findings. Our GGA calculations yielded a direct band at the **K** point of 0.935 eV for pristine monolayer MoTe_2_, which is consistent with other DFT and experimental findings [48,49]. In order to conduct a more comprehensive analysis of the conduction band minimum (CBM) and the valence band maximum (VBM) at the K point, the projected density of states (PDOS) is computed. According to our findings, the VBM and CBM are primarily contributed by Mo 4d and Te 5p states, which undergo a division between their occupied and unoccupied states.

Defects that are most fundamental and frequently manifest in two-dimensional materials are antisites and vacancies. The five optimised point defective structures discussed in this paper are depicted in Figure 1(b–f). These structures include a single Mo vacancy (V_Mo_), a single Te vacancy (V_Te_), double Te vacancy (V_2Te_), and substitutional defects where Te and W atoms replacing Mo atom (Te_Mo_ and W_Mo_). We construct 5 × 5MoTe_2_ supercell to form V_Mo_, V_Te_ , and V_2Te_ defects by removing single Mo atom, single Te atom, and two Te atoms which leads to defect concentrations of 4% for V_Mo_ and V_2Te_ defects and 2% for V_Te_ defect are shown in Figure 1(b–d) respectively. These specific intrinsic point defects are routinely seen in synthetic materials.

By utilising argon plasma treatment, it was possible to generate a defect concentration in the basal plane during experiments [50]. In the range of allowed Te chemical potential, Te-vacancy formation energy is lower than that of Mo-vacancy, indicating that Te vacancies may be generated in the experimental growth environments. This is supported by a previous experimental observation [45] in which Te vacancies were readily formed and frequently detected during vacuum annealing. Additionally, it can be easily modified to display either n-type or p-type transfer characteristics [51].

To assess the vacancy formation in a monolayer MoTe_2_ and a thermodynamically stable defected structure, the formation energy was computed utilising the subsequent formula,

$$E_{f}=\frac{E_{\mathrm{defect}}-E_{\mathrm{pristine}}+\sum_{i}n_{i}\mu_{i}}{N}$$

where $E_{\mathrm{defect}}$ and $E_{\mathrm{pristine}}$ show the respective total energies of the supercell with and without a vacancy defect. Also, $n_{i}$ represents the number of atoms corresponding to element i that has been extracted from the pristine structure. The chemical potential of the element i is represented by $\mu_{i}$. The formula for calculating the formation energy of substitutional Te and W defects in monolayer MoTe_2_ is as follows,

$$E_{f}=\frac{E_{\mathrm{defect}}-\left(E_{\mathrm{pristine}}-\mu_{\mathrm{Mo}}+\sum_{j}n_{j}\mu_{j}\right)}{N}$$

where $\mu_{\mathrm{Mo}}$ and $\mu_{j}$ chemical potentials of Mo and substituted Te (W) atoms. *N* represents the total number of atoms in the supercell.

The formation energies for five types of defects are listed in Table 1. The MoTe_2_ monolayer doped with V_Te_ exhibits the least amount of energy required to form vacancy defects. The V_Mo_ defect exhibits a significantly greater formation energy in comparison to the V_Te_ and V_2Te_ vacancy defects. This observation becomes easily comprehensible when one considers that producing a Mo vacancy necessitates the disruption of twice as many Mo-Te bonds as is necessary to produce a Te vacancy. By replacing Mo atom with Te and W atoms in monolayer MoTe_2_, as shown in Figure 1(e,f), the lowest formation energy has been obtained for W_Mo_ defect. A persistent difficulty is reducing the significant energy requirements linked to creating active surface regions in 2D monolayers by forming vacancies. As an example, the process of forming a single carbon vacancy in graphene requires an energy of more than 7.5 eV [52]. Similarly, in boron-nitride monolayers, a well-known 2D material, the energy required to produce single nitrogen vacancies (V_N_) is roughly 4.5 eV, whereas the energy required for the formation of boron vacancies (V_B_) is around 7 eV [53]. The substantial energy demands associated with the production of flawed 2D materials pose considerable challenges to their extensive use. Nevertheless, MoTe_2_ monolayers provide a hopeful solution for tackling this obstacle, since they possess vacancies that are more easily reached in terms of energy.

**Table 1.** Formation energies and calculated band gap for the structures of pristine and all defective MoTe_2_ monolayers.

Structure	E_f_ (meV/atom)	E_g_ (eV)
Pristine	-	0.935 (direct)
V_Mo_	193.115	-
V_Te_	67.296	0.711 (indirect)
Te_Mo_	156.007	0.113 (direct)
W_Mo_	15.243	0.929 (direct)
V_2Te_	125.651	0.754 (indirect)

**Figure 2.**
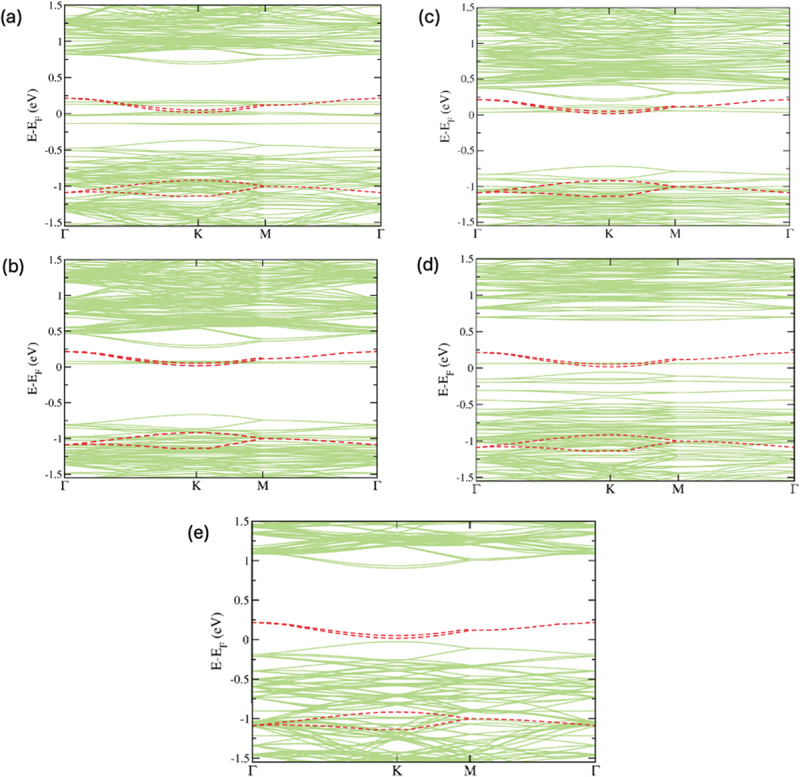
(a) The electronic band structure of monolayer MoTe_2_ with V_Mo_ defect, (b) V_Te_ defect, (c) V_2Te_ defect, (d) Te_Mo_ substitutional defect, and (e) W_Mo_ defect. Fermi level is set to zero. The red dotted lines represent electronic structure of defect free monolayer MoTe_2_.

Creating atomic defects is a frequent method used to regulate the band gap in monolayer MoTe_2_, since they are very effective in modifying the band gap. Furthermore, MoTe_2_ consistently demonstrates ambipolar transfer properties as a result of its narrow band gap and can be readily modified to exhibit *n*- or p-type transfer [54,55]. Figure 2(a–e) display the energy band structures of defective monolayers of MoTe_2_, where the red dotted lines illustrate the electronic structure of a defect-free MoTe_2_ monolayer. Observably, the monolayer MoTe_2_ without defects is a direct semiconductor with a predicted band gap of 0.935 eV. The valence band maximum (VBM) and conduction band minimum (CBM) are situated at the **K** point. Upon the creation of V_Mo_, V_Te_, and V_2Te_ defects, more defect states are created inside the band gap. The introduction of a V_Te_ defect caused a reduction in the band gap by up to 0.711 eV. Nevertheless, the CBM has been relocated to the **Γ** point, while the VBM remains at the **K** point. This results in the transformation of the direct band gap semiconductor monolayer MoTe_2_ into an indirect bandgap semiconductor. Additionally, it is seen that a fresh band emerges underneath the conduction band subsequent to the formation of a vacancy in Te. The presence of a single and double vacancies in MoTe_2_ monolayer creates localised states inside the band gap of the defect-free material. Similarly, we eliminated two Te atoms in order to generate a double Te vacancy, resulting in the V_2Te_ defect. In order to address this defect, the Te atoms are extracted from the upper and lower atomic layers of the pristine monolayer MoTe_2_. We get comparable outcomes using the V_Te_ defect. In Figure 2(c), the band gap decreases to 0.754 eV, causing the direct band gap semiconductor behaviour to transition into an indirect band gap semiconductor. The conduction band minimum (CBM) is located at the **Γ** point, while the valence band maximum (VBM) is located at the **K** point. We notice that the band gap is slightly higher than the band gap of the V_Te_ defect configuration. This is a result of the presence of defect states inside the energy gap. Hence, the manipulation of various point defects allows for the regulation of the band gap in monolayer MoTe_2_.

In the case of a substitutional defect in a monolayer of MoTe_2_, the band gap is greatly lowered to 0.113 eV. However, as demonstrated in Figure 2(d), the material still maintains its direct semiconductor properties. As represented in Figure 2(e), the presence of a W_Mo_ defect in a monolayer of MoTe_2_ results in a small reduction of the direct band gap value to 0.929 eV. Observably, the valence and conduction bands of monolayer MoTe_2_ undergo expansion and shift towards higher energy levels in the presence of all five types of defects. Consequently, these defects confer n-type characteristics onto monolayer MoTe_2_. Recent experimental studies have shown that laser irradiation can be used to create vacancy defects in the MoTe_2_ monolayer [54,55]. When the MoTe_2_ monolayer is exposed to laser irradiation in a vacuum, it exhibits characteristics of an n-doped semiconductor. This is because the Fermi level is positioned above the conduction band and does not change with the gate voltage [54]. In addition, MoTe_2_ crystals can be susceptible to heat damage in the atmosphere due to the relatively low stability of Te atoms. When the temperature reaches 1200 K due to the focused laser, vacancies start to form in the MoTe_2_ crystal. This happens because the temperature exceeds the threshold of 400 K for producing vacancies in TMDCs [55]. According to previously reported works [54,55], using laser irradiation is a proven method to achieve n-type doping in MoTe_2_ crystal. Our calculations also have shown the presence of a donor level near the conduction band in MoTe_2_.

**Figure 3.**
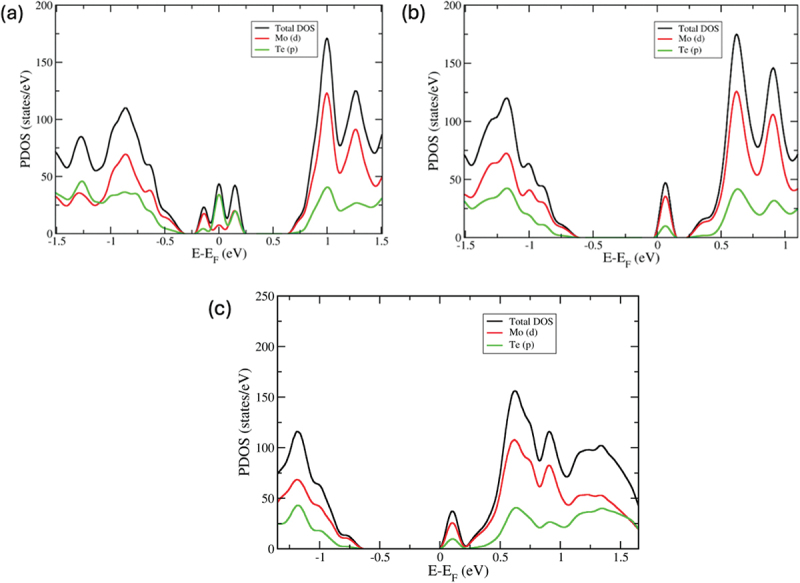
The total and projected density of states for (a) V_Mo_ defect, (b) V_Te_ defect, and (c) V_2Te_ defect. The fermi level is set to zero.

**Figure 4.**
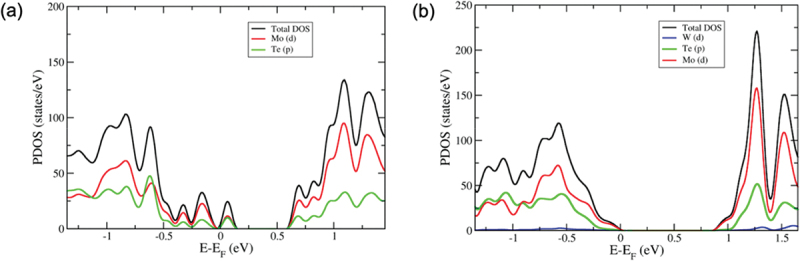
(a) The total and projected density of states for (a) Te_Mo_ defect and (b) W_Te_ defect. The fermi level is set to zero.

The total density of states (TDOS) and projected density of states (PDOS) are computed, as illustrated in Figures 3 and 4, in order to elucidate the alterations in the electronic structure brought about by the emergence of these five defect types. Figure 3(a) displays the TDOS and PDOS of monolayer MoTe_2_ with a V_Mo_ vacancy. It is clear that the presence of a Mo vacancy in the monolayer MoTe_2_ significantly alters the density of states. The valence and conduction bands of monolayer MoTe_2_ have undergone an expansion towards higher energy. The V_Mo_ defect produces three peaks near the Fermi level, indicating that the Mo vacancy creates acceptor-like levels in the band gap, resulting in a higher p-type orientation for this defect. The band gap states primarily arise from the p states of the six-nearest neighbour Te atoms surrounding the Mo vacancy, with some contribution from the d states of the Mo atoms. It has been observed that the presence of both single and double Te vacancies leads to a reduction in the band gap due to the emergence of new states near the conduction band minimum. The states primarily arise from the contributions of d molybdenum orbitals, as shown in Figure 3(b,c). Through the formation of vacancies, the electronic properties can be modulated. In addition, the energy levels caused by the absence of Te atoms are higher than the Fermi level, resulting in the formation of levels that act as donors.

In Figure 4(a), it is observed that the VBM is primarily influenced by Mo d orbitals, while the CBM is influenced by both Mo d and Te p orbitals in the PDOS of Te_Mo_ substitutional defects. When the Mo atom is replaced with Te in the pristine monolayer MoTe_2_, there is a noticeable alteration in the band gap. It is evident that the electronic properties and chemical activity of the molybdenum telluride monolayer are extremely responsive to substitutional defects. In Figure 4(b), the PDOS for the W_Mo_ defect in monolayer MoTe_2_ is displayed. The dominant formation of band edge states in monolayer MoTe_2_ is attributed to the d orbitals of Mo in the W_Mo_ defect. The contributions are most significant near the energy gap, with the one from Te p orbitals appearing approximately 1 eV below the Fermi level. There appears to be a minor involvement of the W d orbital in the CBM. The defect state is created above the Fermi level. The band gap value of defect-free monolayer MoTe_2_ is slightly reduced when W_Mo_ defect is introduced.

**Figure 5.**
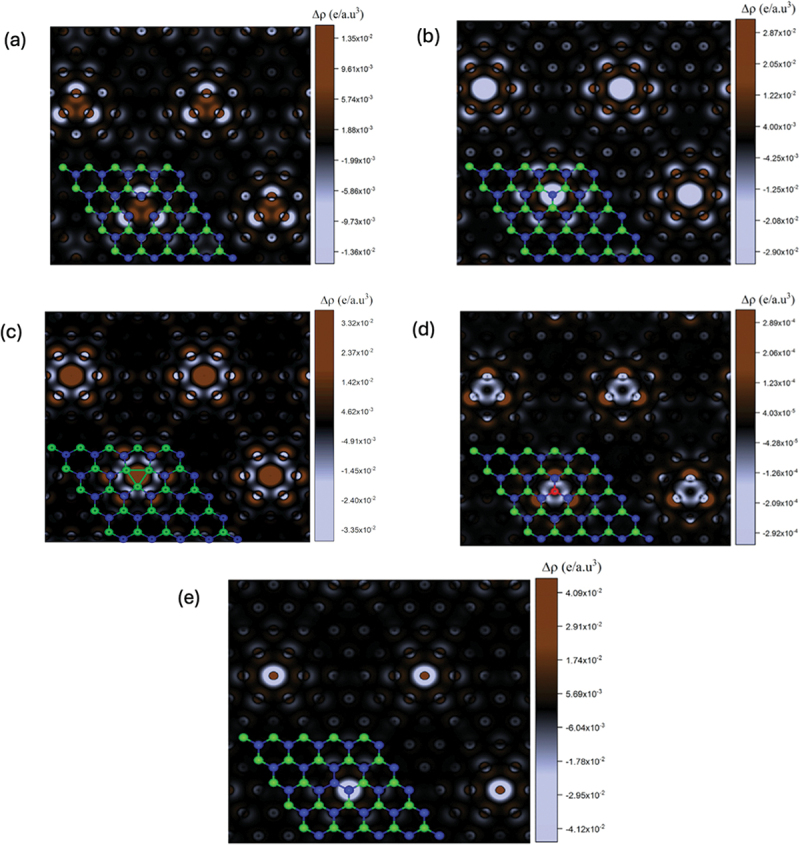
Charge density difference for (a) V_Mo_ defect, (b) V_Te_ defect, (c) V_2Te_ defect, (d) W_Mo_ defect, and (e) Te_Mo_ defect.

As demonstrated in the previous section, the presence of defects significantly alters the electronic levels of the MoTe_2_ monolayers. Figure 5(a–e) illustrates the calculated charge density differences for the various defects examined, providing valuable insight into the changes in electron density caused by vacancies and substitutional defects. Figure 5(a–c) demonstrates the absence of charge in the locations where Mo and Te atoms are eliminated, indicating vacancy defects. As a result, there is a significant reorganisation of charge around the vacancies. It is evident from Figure 5(a) that the Te atoms surrounding the Mo vacancy have acquired a higher charge compared to the rest. Two Te atoms surrounding the Mo atom adjacent to the defected region experienced an increase in charge in the case of the V_Te_ and V_2Te_ defects. The charge accumulation is reduced when comparing those Mo vacancy defects. Figure 5(d,e) display the variations in charge density for the substitutional W_Mo_ and Te_Mo_ defects. Figure 5(d) demonstrates that the substituted W atom provides a greater charge to the surrounding Te atoms compared to the V_Mo_ defect. When a Mo atom is replaced with a Te atom, the charge is transferred to the Te atom surrounding the Te_Mo_ defect. The Te atom in question receives a charge from the molybdenum atoms and is then substituted by another Te atom, as depicted in Figure 5(e). It has been demonstrated that there is a significant rearrangement of charge surrounding the substitutional Te atom. The electron density around the Te atoms in defected monolayer MoTe_2_ remains largely unaffected.

## Conclusion

Our present study explores the electronic properties of monolayer MoTe_2_, a material known for its remarkable electronic characteristics, and its potential applications in opto-electronic and nano-electronic fields. Point defects are quite common during the growth of MoTe_2_ and have a significant impact on its properties. Using first-principles calculations based on DFT, our investigation delves into the electronic properties of these defects, including point defects. Based on our results, it appears that the presence of point defects leads to the development of n-type characteristics as the Fermi-level close to the conduction band near the band edges. Our research offers valuable theoretical support for defect engineering in MoTe_2_ monolayers and other 2D materials. It plays a crucial role in the development of nanoscale devices with the desired functionality. Our findings suggest that vacancy defects have the potential to significantly impact the electronic properties of monolayer MoTe_2_, positioning it as an exciting prospect for future electronics applications. Our work emphasises the significance of comprehending how vacancy defects impact electronic properties when selecting materials for manipulation in different synthesis and processing conditions. We found that the formation energy of substitutional defects in monolayer MoTe_2_ is lower than that of Mo-vacancy. This suggests that Te vacancies could potentially be created in the experimental growth environments. According to our study, MoTe_2_ monolayers offer a potential solution to decrease the high energy demands associated with creating active surface areas in 2D monolayers through the formation of vacancies.

Our study suggests that by manipulating atomic defects, the band gap in monolayer MoTe_2_ can be controlled, resulting in adjustments to the valence and conduction bands. Defects in the material have a significant impact on its electronic structure, resulting in the emergence of n-type characteristics. The valence and conduction bands of MoTe_2_ experience an expansion and move towards higher energy levels. The presence of the V_Mo_ defect leads to the appearance of three peaks close to the Fermi level. This suggests that the Mo vacancy generates levels with characteristics similar to acceptors within the band gap. The band gap states mainly originate from the p states of the six-nearest neighbour Te atoms surrounding the Mo vacancy, along with a slight contribution from the d states of the Mo atoms. Substitutional defects have a significant impact on the electronic properties and chemical activity of the MoTe_2_ monolayer. Examining the charge density differences for the different defects offers valuable insight into how vacancies and substitutional defects affect the electron density.
